# Older adults’ vaccine hesitancy: psychosocial predictors of influenza, pneumococcal, & shingles vaccine uptake

**DOI:** 10.1093/geroni/igaa057.3358

**Published:** 2020-12-16

**Authors:** Louise Brown Nicholls, Allyson Gallant, Nicola Cogan, Susan Rasmussen, David Young, Lynn Williams

**Affiliations:** University of Strathclyde, Glasgow, United Kingdom

## Abstract

Influenza, pneumococcal disease, and shingles are more prevalent in older people, with this group having an increased risk of developing severe illnesses and complications. These illnesses are preventable via vaccination, but uptake of these vaccines is low and decreasing year-on-year. However, little research has focused on understanding the reasons behind vaccine hesitancy in older adults. We implemented a cross-sectional survey to determine the self-reported vaccination behaviours of 372 UK-based adults aged 65-92 years. We assessed previous uptake and future intention to receive the influenza, pneumococcal, and shingles vaccines. Participants also self-reported their health and socio-demographic data, and completed two scales measuring the psychological factors associated with vaccination behaviour (5C and VAX scales). Self-reported daily functioning, cognitive ability, and social support were also assessed. Considerably more participants had received the influenza vaccine in the last 12 months (83.6%), relative to having ever received the pneumococcal (60.2%) and shingles vaccines (58.9%). Multivariate logistic regression analyses showed that a lower sense of collective responsibility independently predicted lack of uptake of all three vaccines in this population. Greater calculation of the disease/vaccination risk and preference for natural immunity also predicted not getting the influenza vaccine. For both the pneumococcal and shingles vaccines, concerns about profiteering predicted lack of uptake. Therefore, more understanding of vaccine benefits and disease risks may be required for these vaccines. Additional qualitative data generally supported these findings, which can contribute to future intervention development and research targeted at more diverse groups (e.g. older adults with cognitive impairments).

